# High olive oil diets enhance cervical tumour growth in mice: transcriptome analysis for potential candidate genes and pathways

**DOI:** 10.1186/s12944-019-1023-6

**Published:** 2019-03-28

**Authors:** Xiaoyu Zhang, Ping Yang, Xuan Luo, Chunxiao Su, Yao Chen, Lei Zhao, Li Wei, Han Zeng, Zac Varghese, John F. Moorhead, Xiong Z. Ruan, Yaxi Chen

**Affiliations:** 1 Centre for Lipid Research & Key Laboratory of Molecular Biology for Infectious Diseases (Ministry of Education), Institute for Viral Hepatitis, Department of Infectious Diseases, The Second Affiliated Hospital, Chongqing Medical University, Chongqing, 400016 China; 2 John Moorhead Research Laboratory, Centre for Nephrology, University College London Medical School, Royal Free Campus, University College London, NW3 2PF, London, UK

**Keywords:** High olive oil diet, Cervical cancer, Transcriptome analysis; differentially expressed genes

## Abstract

**Background:**

Numerous epidemiologic studies have found a close association between obesity and cancer. Dietary fat is a fundamental contributor to obesity and is a risk factor for cancer. Thus far, the impact of dietary olive oil on cancer development remains inconclusive, and little is known about its underlying mechanisms.

**Methods:**

Nude mouse xenograft models were used to examine the effects of high olive oil diet feeding on cervical cancer (CC) development and progression. Cell proliferation, migration and invasion were observed by the methods of EdU incorporation, Wound healing and Transwell assay, separately. RNA-sequencing technology and comprehensive bioinformatics analyses were used to elucidate the molecular processes regulated by dietary fat. Differentially expressed genes (DEGs) were identified and were functionally analyzed by Gene Ontology (GO), Kyoto Enrichment of Genes and Genomes (KEGG). Then, protein–protein interaction (PPI) network and sub-PPI network analyses were conducted using the STRING database and Cytoscape software.

**Results:**

A high olive oil diet aggravated tumourigenesis in an experimental xenograft model of CC. Oleic acid, the main ingredient of olive oil, promoted cell growth and migration in vitro. Transcriptome sequencing analysis of xenograft tumour tissues was then performed to elucidate the regulation of molecular events regulated by dietary fat. Dietary olive oil induced 648 DEGs, comprising 155 up-regulated DEGs and 493 down-regulated DEGs. GO and pathway enrichment analysis revealed that some of the DEGs including EGR1 and FOXN2 were involved in the transcription regulation and others, including TGFB2 and COL4A3 in cell proliferation. The 15 most strongly associated DEGs were selected from the PPI network and hub genes including JUN, TIMP3, OAS1, OASL and EGR1 were confirmed by real-time quantitative PCR analysis.

**Conclusions:**

Our study suggests that a high olive oil diet aggravates CC progression in vivo and in vitro. We provide clues to build a potential link between dietary fat and cancerogenesis and identify areas requiring further investigation.

**Electronic supplementary material:**

The online version of this article (10.1186/s12944-019-1023-6) contains supplementary material, which is available to authorized users.

## Background

Cervical cancer (CC) is the fourth leading cause of cancer-related death among women worldwide with approximately 527,600 new cases each year [1] with about 89% of deaths occurring in less developed countries [2]. The oncogenic types of human papillomavirus (HPV) persistent infection are the most common cause of CC [3]. However, progression from high-risk HPV-positive premalignant lesions to malignant carcinoma seldom occurs [4], indicating additional contributors are required to promote CC’s malignant transformation and progression.

Obesity and lifestyle factors, such as smoking, nutrition and physical activity, are considered risk factors in the development of CC. In recent years, a large body of emerging evidence has elucidated a strong association between obesity and CC morbidity and mortality [5–7]. Dietary fat is a fundamental contributor to obesity [8, 9] and is one of the potential factors thought to link obesity and CC. Accumulating evidence has demonstrated that dietary lipids are associated with various malignant tumours, such as breast cancer, colorectal cancer, pancreatic cancer, and prostate cancer [10–12]. Additionally, recent evidence has suggested that both amount and type of dietary fat are important in the cancer aetiology [13].

Olive oil, a common ingredient of Mediterranean diet [14], has attracted much attention, especially in the last few years. Numerous epidemiological studies have reported that cancer occurrence in the Mediterranean was lower, especially involving in the endometrium, breast, skin, intestine and prostate, suggesting protective effects of olive oil [14–17]. Olive oil exhibits beneficial effects due to various antioxidants, including Vitamin E and phenolic compounds such as tyrosol, hydroxytyrosol, and oleuropein. Moreover, it has been demonstrated that, besides antioxidant ability, olive oil exerts anti-inflammatory and anti-neoplastic activities [18, 19]. It displays anti-tumourigenic properties by promoting apoptosis and cell cycle arrest in various cancers, such as lung, oesophageal and colon cancers [20]. However, contrasting results have been reported to show olive oil’s tumour-enhancing effects. Oleic acid (OA), which constitutes 70–80% of olive oil, promotes cell proliferation and migration in highly metastatic gastric and breast cancer cells in an AMPK-dependent manner [21]. A large population-based case-control study of OA indicated that it may increase the risk of pancreatic cancer [22]. Additionally, a high OA diet exhibits a tumour-enhancing effect in rats by modulating epigenetic patterns [23]. Hence more work is needed to clarify the association between dietary olive oil and cancer.

To assist in clarifying disparate outcomes, we examined the effects of high olive oil diet feeding on CC development and progression using nude mouse xenograft models. We performed a comparative transcription analysis of xenograft tumour samples using RNA-seq technology to detect the differential transcriptomic characteristics. Our findings may provide novel insights into understanding the underlying molecular mechanism between dietary lipids and CC.

## Materials and methods

### Cell culture and mouse xenograft models

CC HeLa cells were cultured in high-glucose Dulbecco’s modified Eagle’s medium (DMEM) supplemented with 10% foetal bovine serum (FBS) and 1% penicillin-streptomycin. The cells were grown in a humidified atmosphere with 5% CO_2_ at 37 °C and were subcultured as needed using trypsin-EDTA. Animal care and experimental procedures were approved by the Animal Care Committees at Chongqing Medical University. Four-week-old athymic male BALB/c-nude mice were fed a control diet (CD; 10 kcal % fat), or a high olive oil diet (OD; 45 kcal % fat) ad libitum until the end of the study (*n* = 5). The detailed nutritional profiles of the diets are presented in Additional file 1: Table S1. A total of 5 × 10^6^ cells in 200 μl PBS was injected subcutaneously into the left flank of the animals. The tumour dimensions were measured every 4 days, and the volumes were calculated by the standard formula: length×width^2^/2. All the animals were sacrificed 6 weeks after injection, and the tumours were excised, weighed and treated for histological examination.

### Histology and immunohistochemistry (IHC)

Xenograft samples were fixed in 4% paraformaldehyde overnight at 4 °C. Subsequently, after dehydration in ethanol, clearing in xylene, and embedding in paraffin, 5-μm-thick histological sections were obtained. To evaluate tumour heterogeneity and pathological grade, the tissue sections were stained with haematoxylin and eosin (H&E). The IHC procedure was applied according to the instructions of a commercial kit (ZsBio, China). The sections were incubated with anti-proliferating cell nuclear antigen (PCNA) primary antibodies (1:8000; CST, USA) at 4 °C overnight. The IHC results were analysed using a microscope by two separate researchers at 400 × magnification in five randomly selected representative fields.

### Cell proliferation assays

For growth curves, HeLa cells were seeded in 96-well plates treated with or without 10 μM OA and were imaged in an IncuCyte (Essen, USA) automated incubator microscope. Pictures were taken every 4 h., and cell confluence was calculated per well using an associated software algorithm. DNA synthesis was determined using a Cell-Light EdU Apollo 643 In Vitro Imaging Kit (RiboBio, China) according to the manufacturer’s instructions. Briefly, the cells were incubated with 50 μM EdU for 1 h. before fixation with 4% paraformaldehyde, permeabilization in 0.3% Triton X-100, and EdU staining. The EdU-positive cells were counted randomly in five fields under a microscope (× 100).

### Wound-healing and Transwell migration assays

Wound-healing and Transwell migration assays were performed to evaluate the parallel and vertical migration ability of the cells, respectively. For the wound-healing assay, HeLa cells were plated on 24-well plates, scratched with a pipette tip, and washed with PBS to remove cell debris. Wound closure was observed and photographed at 0, 24 and 48 h. For the Transwell assay, 2 × 10^4^ cells in 100 μl of serum-free medium with or without 10 μM OA were loaded into the upper chamber. Medium containing 10% FBS was added to the lower chamber. After incubation, cells that adhered to the lower surface of the filter were fixed, stained and photographed. Migration was determined by counting the cells in five randomly selected visual fields.

### RNA extraction, cDNA construction, and sequencing

Total RNA was prepared from CD-fed mouse xenograft samples and OD-fed mouse xenograft samples using TRIzol reagent (Takara, Japan) according to the manufacturer’s protocol, after which the concentration was measured using the NanoDrop 2000 system (Thermo Fisher, USA). During the quality control steps, the Agilent 2200 Bioanalyser system (Agilent, USA) was used for qualification and quantification of the RNA samples. The RNA extracted with the following criteria was subjected to RNA-Seq analysis: RNA integrity number, ≥7; OD260/OD280, 1.9–2.1; 28S/18S ratio, > 1.8. Next, cDNA was synthesized using the Ion Total RNA-Seq Kit v2 (Life Technologies, USA) according to the manufacturer’s instructions. After library construction, each library preparation was sequenced using the Illumina HiSeq™ 2000 system (Illumina, USA).

### Identification of DEGs

After sequencing, raw reads were generated. Next, clean reads were obtained after filtering low-quality reads or reads with an adapter sequence. Further analyses were performed based on those clean reads, and they were mapped to the reference genome in the Ensemble database using Misplacing software [24]. To quantify the gene expression levels, a reliable method considering the gene length for the read counts and effects of the sequencing depth was adopted. Thus, the expression level of each gene was normalized by the reads per kilobase per million (RPKM) [25]. The EBSeq package was implemented to screen DEGs [26], and they were filtered employing a fold-change (FC) method and false discovery rate (FDR) correction, exhibiting an FC > 2 along with FDR < 0.05.

### Gene ontology (GO) and pathway enrichment analysis

To analyse the DEGs at the functional level, GO enrichment analysis and Kyoto Encyclopedia of Genes and Genomes (KEGG) pathway analysis of the DEGs were conducted. Here, the online tool adopted in this study for GO and KEGG analysis was Database for Annotation, Visualization and Integrated Discovery (DAVID, https://david.ncifcrf.gov/home.jsp) [27]. We submitted the DEGs list into it and selected *Homo sapiens* in the species column. Finally, the GO terms or KEGG pathways with the cut-off criteria (*P* < 0.05) were chosen as the enriched function of the DEGs.

### Protein-protein interaction (PPI) network and hub gene analysis

To further investigate the molecular mechanism of CC, all DEGs were used to construct the PPI network using the biological online database tool (Search Tool for the Retrieval of Interacting Genes, STRING, http://string-db.org) [28] to determine and predict the interaction among them. A combined score > 0.7 (high confidence score) was considered significant, and then the PPI network was visualized using Cytoscape software (Version 3.5.1) [29]. To evaluate the importance of nodes in the PPI network, the degree centrality of nodes was calculated and utilized in the present study [30] using the CytoNCA plugin [31] in Cytoscape software. The hub genes/proteins, a small number of crucial nodes for the protein interactions in the PPI network, were chosen with a centrality degree > 8. Because a higher k-core score means a more topological central location, subnetworks in the PPI network were explored by k-core scoring using the MCODE plugin in Cytoscape software, and significant subnetworks with a k-core > 6 were considered potential core regulatory networks.

### Real-time quantitative PCR (RT-qPCR)

To validate the RNA-Seq results, cDNA was synthesized by reverse transcription using 1 μg of the original RNA sample as described previously. Next, the cDNA products were subjected to 2-step PCR amplification. RT-qPCR using the SYBR Green method was used to detect the gene expression level. β-Actin was chosen as the reference gene, and the expression levels were computed according to the 2-ΔΔCt method. The primer sequences are provided in Additional file 2: Table S2.

### Statistical analysis

The data were represented as the means ± SEM. Differences between the two groups were assessed by Student’s t-test, analysed using the SPSS 22.0 software and considered significant at *P* < 0.05.

## Results

### High olive oil diet promotes tumour growth in an experimental CC model

**Fig. 1:**
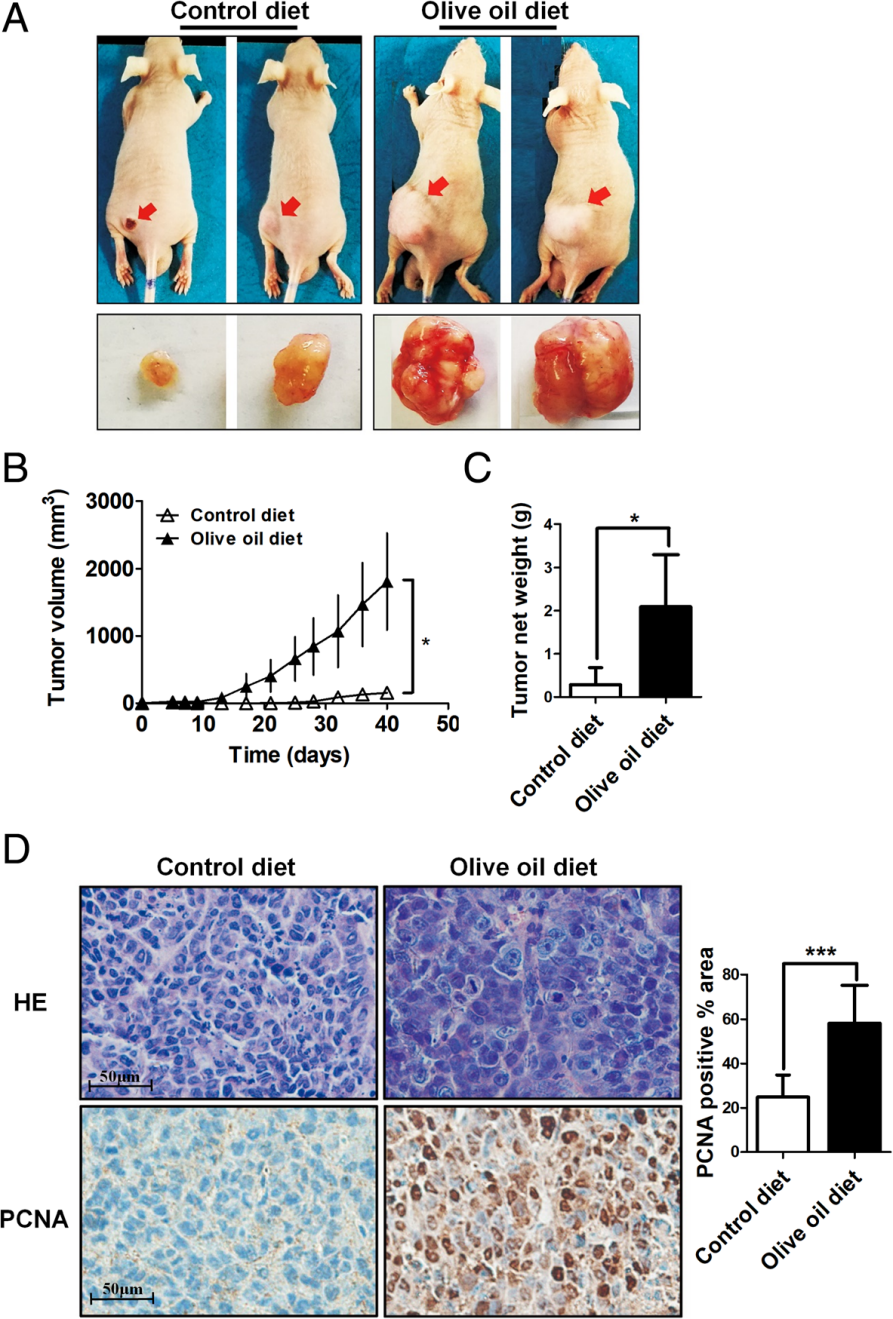
High olive oil diet feeding promotes the growth of xenografts in nude mice. HeLa cells were injected subcutaneously into 4-week-old nude mice fed a control or an olive oil diet ad libitum. At 6 weeks after implantation, the animals were sacrificed, and the tumour masses were excised. **a** Representative graph of tumours formed by the implantation of HeLa cells under different diets. **b** Analysis of tumour growth curves over a 6-week time course. **c** Tumour weights in different diet groups of nude mice. **d** Top, H&E-stained sections of xenografts. Bottom, Immunohistochemical staining of tumour sectios using an anti-PCNA antibody. Original magnification, × 400. Right, quantification of the percentage of the PCNA-positive area. The data were statistically analysed using Student’s t-test, and values are shown as the means ± SD. *P < 0.05, ***P < 0.001

HeLa cells were implanted subcutaneously to CD- and OD-fed nude mice to address the effects of dietary olive oil on tumourigenesis. Mice were sacrificed on day 40, and the tumour xenografts were dissected, weighed and photographed (Fig. 1a). Tumour growth over a 6-week time course was increased significantly in the OD group vs. the CD group (Fig. 1b). High olive oil diet also markedly increased the weight of the xenografts by more than 6-fold (Fig. 1c). Furthermore, OD group xenograft tissues exhibited nuclear hyperchromasia and increased nucleus-to-cytoplasmic ratios, representing poor differentiation and higher heterogeneity (Fig. 1d, upper). Likewise, IHC analyses further uncovered a significant increase in PCNA-positive cells in the OD group (Fig. 1d, lower). These data indicated that high olive oil feeding in nude mice can promote tumour cell growth in vivo.

### OA promotes CC cell growth and migration in vitro

**Fig. 2:**
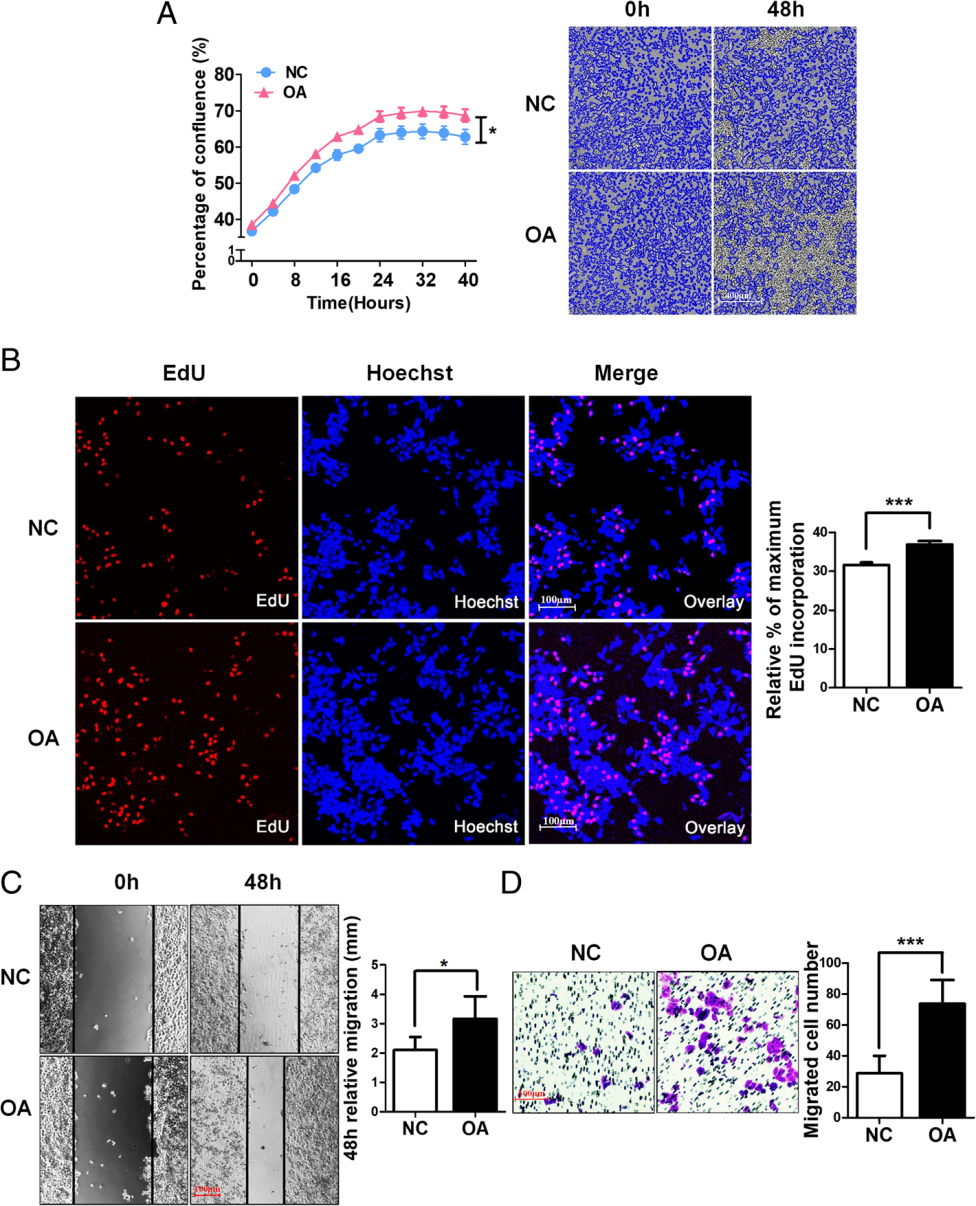
OA accelerates HeLa cell proliferation and migration. **a** Growth curves for HeLa cells treated with or without OA were analysed using an IncuCyte incubator microscope (the mean confluence values were compared at 48 h.; *n* = 5). **b** DNA synthesis of HeLa cells subjected to the EdU incorporation assay. EdU staining (red). Cell nuclei were stained with Hoechst33342 (blue). The quantification of EdU-positive cells was conducted macroscopically and was expressed as a percentage relative to the control cells. **c** OA promotes scratch-induced migration of HeLa cells. Representative phase contrast micrographs of cells treated with or without OA for 0 h. and 48 h.. Relative migration distances are calculated representing scratch closure after 48 h. compared with the initial distances. **d** Transwell assays were performed to investigate the effects of OA on the migration ability of HeLa cells. Quantitative analysis of migration experiments demonstrated that OA promotes HeLa cell migration compared with NC. *P < 0.05, ***P < 0.001

Because OA (more than 70% enriched in olive oil) is the most important functional nutrient component of olive oil, we next explored the effects of OA on the malignant phenotype of tumour cells in vitro. First, using the IncuCyte-automated incubator-microscope system, we observed a clear increase in cell proliferation following 10 μM OA treatment (Fig. 2a). Next, the EdU assay was used to detect the possible change in DNA synthesis. The results showed that the EdU-positive cells were increased by OA treatment (*P* < 0.05) compared with the negative control (NC) group (Fig. 2b). Additionally, to investigate the influence of OA on the migration of HeLa cells in vitro, the wound-healing assay and Transwell assay were performed. The results showed that OA treatment markedly enhanced the cell migration ability in HeLa cells (Fig. 2c, d).

### Differentially expressed mRNAs under high olive oil diet feeding

**Fig. 3:**
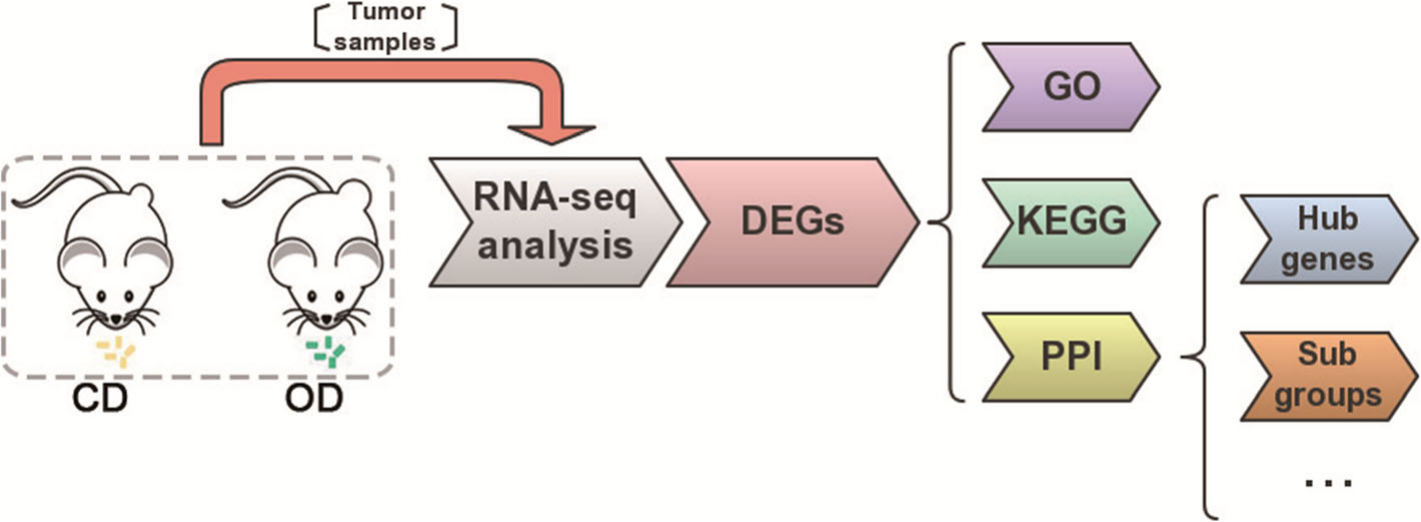
Flowchart of RNA-seq transcriptomic bioinformatics analysis in xenograft tumour samples representing different dietary treatment groups

**Fig. 4:**
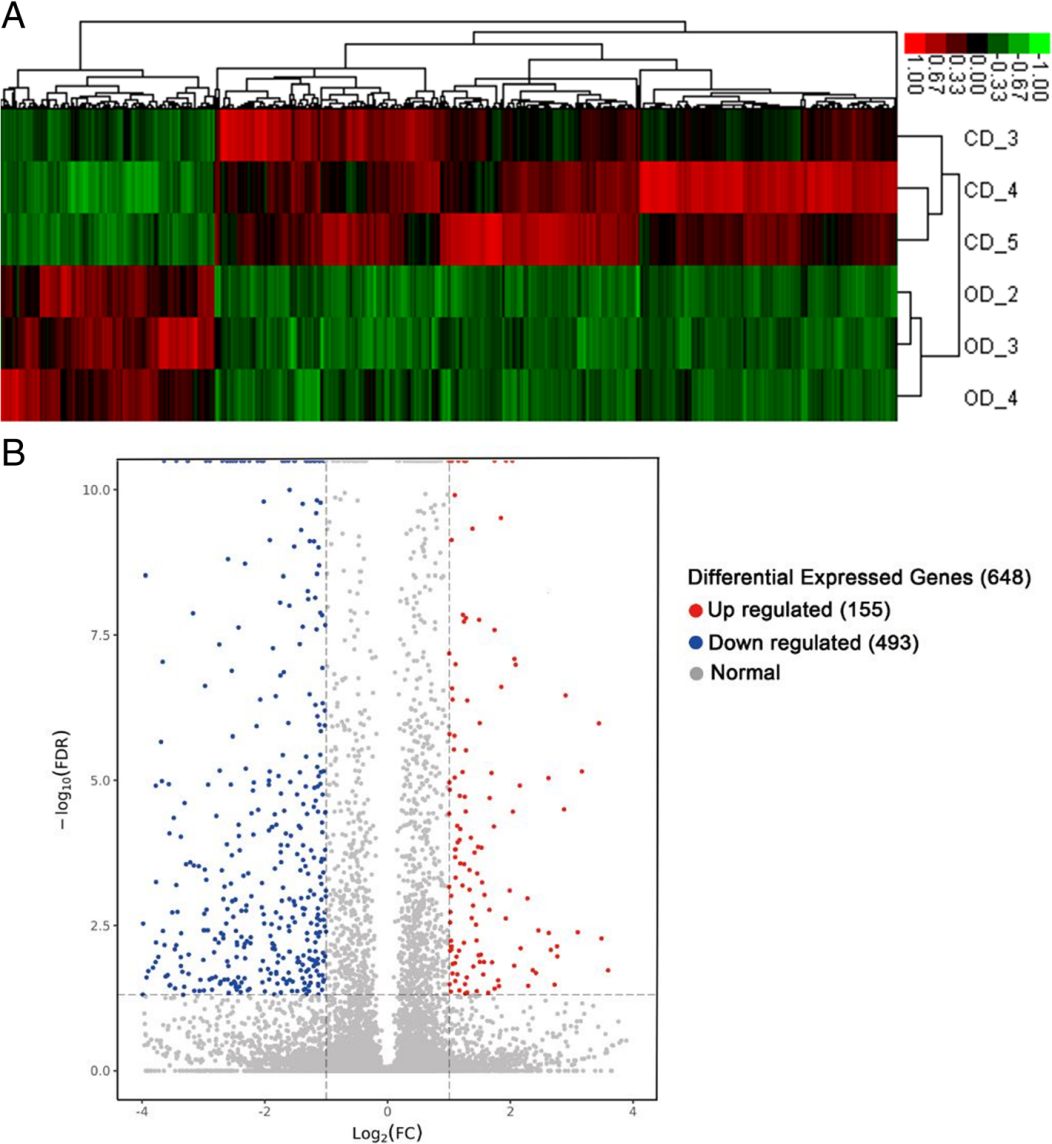
Heat map and volcano plots showed the expression profiles of mRNAs under two diet conditions. **a** Heat map for the DEGs. Each row represents a tissue sample (total 6 samples); each column represents a single gene. The gradual colour ranged from red to green represented the mRNA expression changing from up-regulation to down-regulation. **b** The volcano plot showed significantly changed mRNAs with FDR < 0.05 and |log2FC (fold change)| ≥ 1. The red-marked nodes represented up-regulated genes (155), the blue ones represented down-regulated genes (493), and the grey ones showed no significance

To identify the changes in the transcriptional profiles under different nutritive conditions, we selected three xenograft tumour samples from two groups for RNA-seq analysis. The gene expression level was computed and normalized by RPKM. Subsequently, analysis of DEGs was performed as shown in Fig. 3. Based on the cut-off criteria, in total, 648 genes displayed differential expression in the OD group, including 155 up-regulated DEGs and 493 down-regulated DEGs, compared with the control diet. Hierarchical cluster analysis showing systematic variations of the samples revealed that the DEGs could be utilized to accurately distinguish the OD samples from the CD samples (Fig. 4a). Volcano plots were used to assess gene expression variation between the groups (Fig. 4b). These data indicated that the expression of mRNAs under the high olive oil dietary condition differs from that under the control dietary condition.

### Functional annotation and pathway enrichment of DEGs

**Fig. 5:**
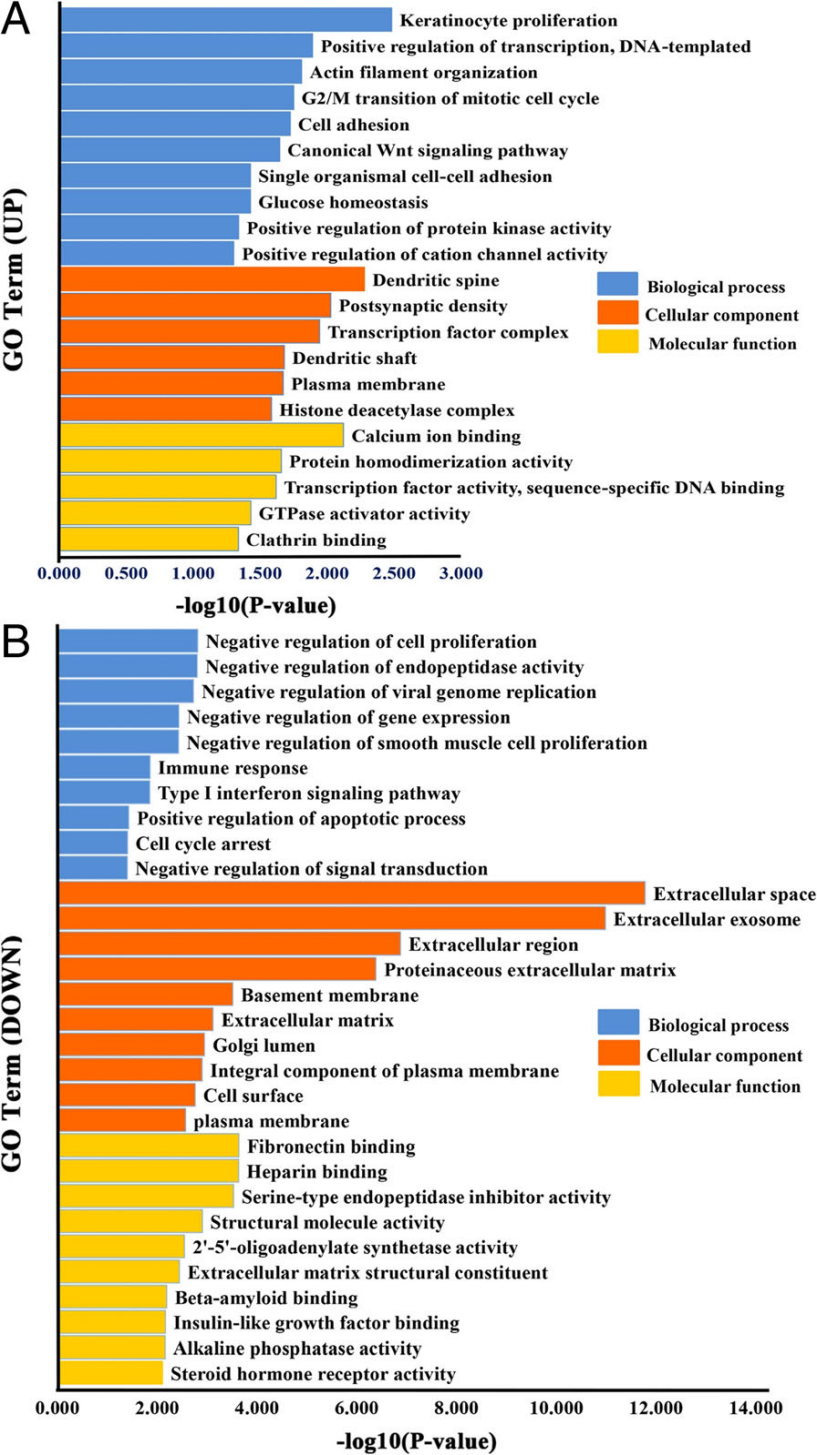
GO analysis of the DEGs. The GO categories include BP, CC and MF, respectively. Highly enriched GO terms of up- and down-regulated mRNAs from the DAVID database with 10 enrichment scores. **a** Up-regulated DEGs. **b** Down- regulated DEGs

Next, to obtain a more comprehensive understanding and identify the functional categories of the DEGs, the data were clustered through GO analysis in DAVID. The significantly enriched GO terms with a *P* value < 0.05 that were divided into biological process (BP), cellular component (CC) and molecular function (MF) ontologies are illustrated in Fig. 5. Regarding the BP ontology, comparing the OD with the CD groups, the highly enriched GO terms of up-regulated DEGs were mainly related to positive regulation of transcription (10 genes), G2/M transition of the mitotic cell cycle (5 genes) and cell adhesion (9 genes). Consistently, the down-regulated DEGs were involved in significant subcategories, including negative regulation of cell proliferation (20 genes), negative regulation of viral genome replication (6 genes) and immune response (18 genes). In the CC ontology, we found that the majority of up-regulated DEGs were associated with subcategory named plasma membrane (40 genes) whereas down-regulated DEGs were involved in multiple cell components such as extracellular space (72 genes), extracellular exosome (114 genes) and plasma membrane (114 genes). In the MF ontology of all DEGs, the binding-related items constituted most of the enriched GO categories, including calcium ion binding (12 genes), clathrin binding (3 genes), fibronectin binding (6 genes), and heparin binding (13 genes). Furthermore, according to the KEGG pathway analysis results (data not shown), the up-regulated DEGs were found to be mostly enriched in the Wnt signalling pathway (4 genes), while the dysfunctional pathways enriched in down-regulated DEGs included antigen processing and presentation (7 genes), the P53 signalling pathway (6 genes), and phagosome (10 genes).

### PPI network analysis

**Fig. 6:**
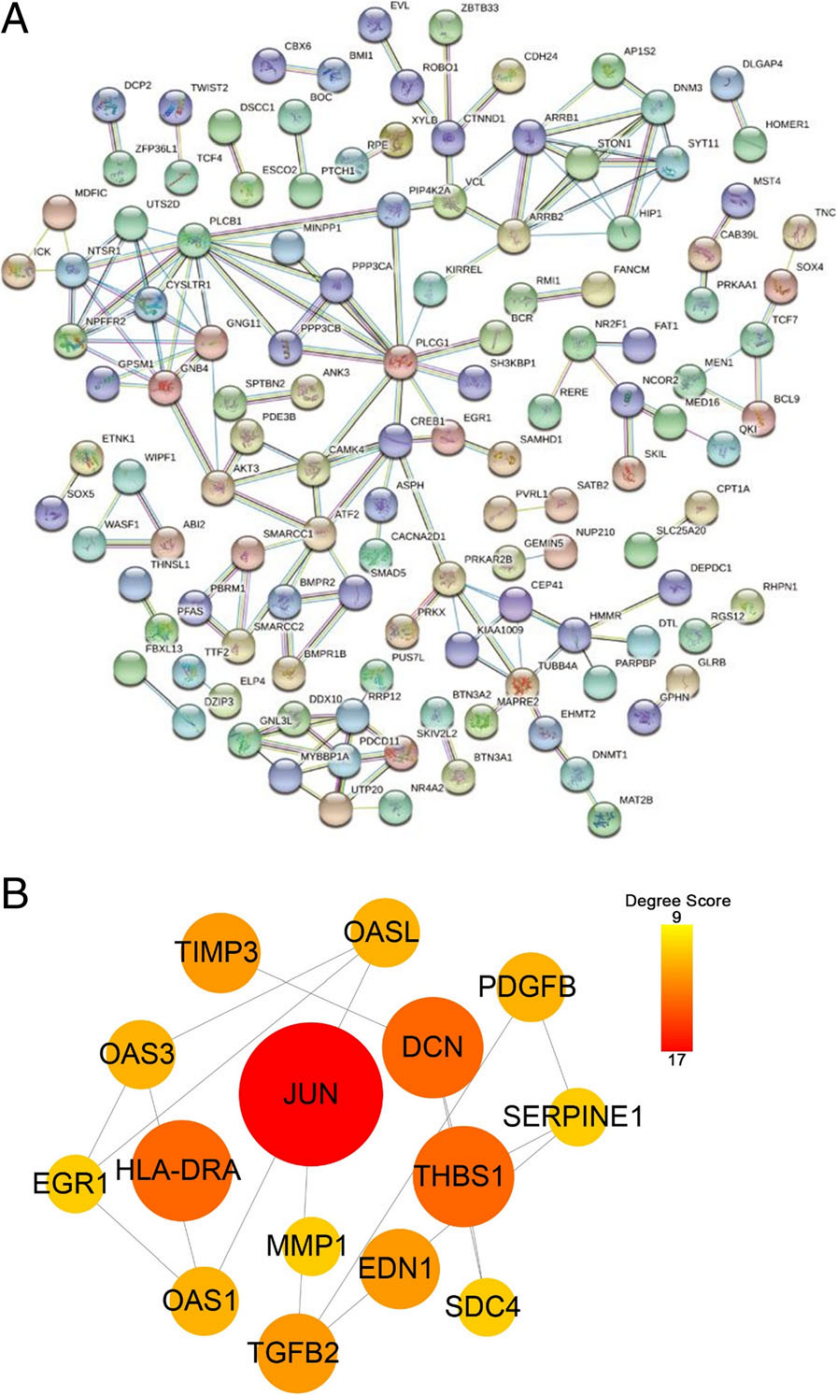
PPI network of DEGs using the STRING. **a** STRING database constructed the network (high confidence score more than 0.7) based on protein-protein interactions, including experiments, co-expression and gene fusion. **b** The 15 genes calculated by degree centrality with a degree score ranging from 9 to 17 were selected as Hub genes; The size and colour change of the nodes denoted the level of degree score

**Fig. 7:**
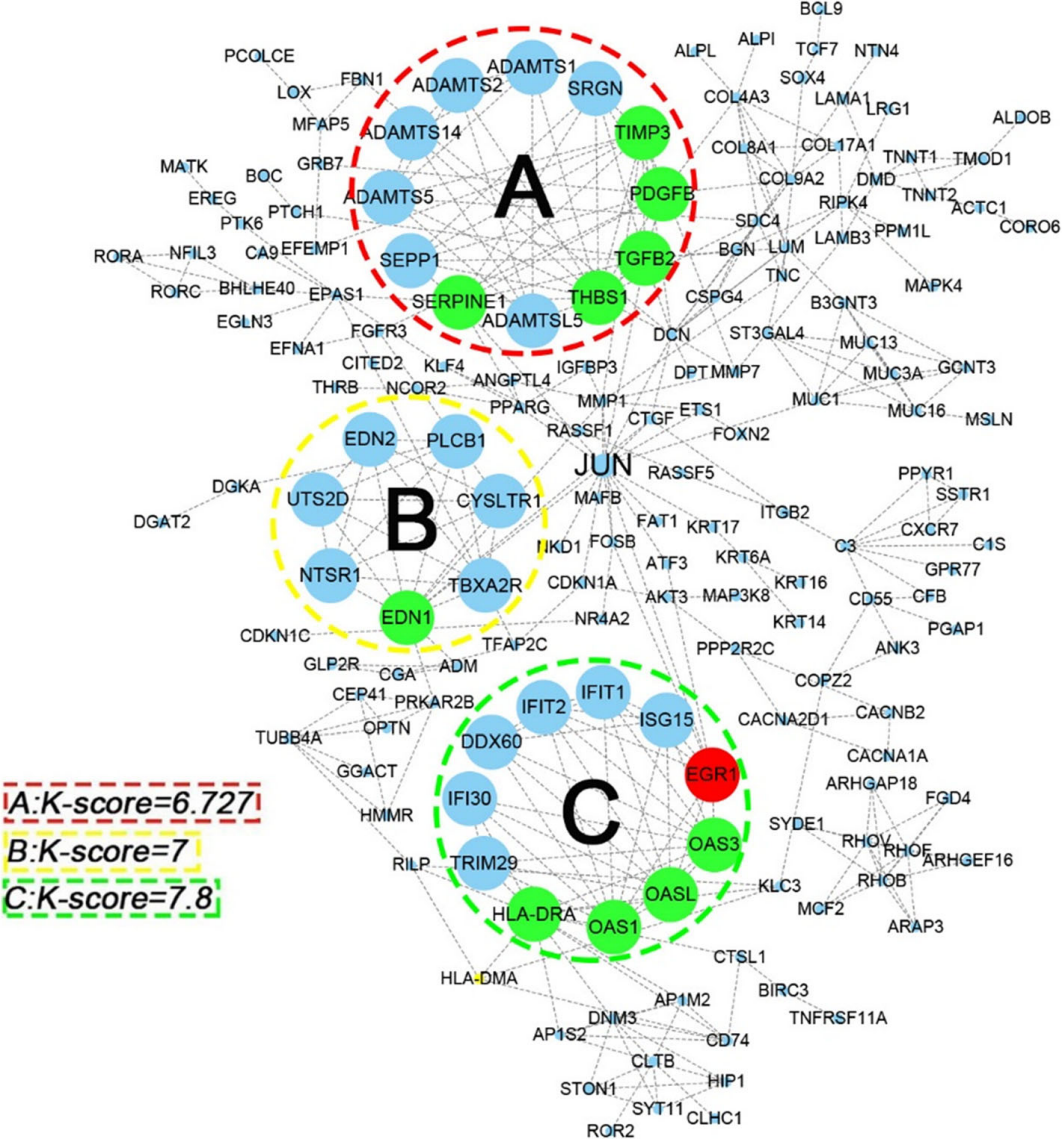
Top 3 modules from the PPI network. The significant 3 clusters marked **a**, **b** and **c**, respectively, identified from the PPI network with k-core > 6, were regarded as topological central networks. Red-marked nodes represented up-regulated hub genes participating in three sub-networks, and the green marked ones represented down-regulated genes

To further investigate the molecular mechanism of the high olive oil diet-promoted tumour growth and interactive relationships among all DEGs, we mapped the 648 DEGs to the STRING database, and validated interactions with a combined score greater than 0.7 (high confidence) were selected to construct a PPI network. The PPI network consisted of 565 nodes and 401 interactions (Fig. 6a). In the PPI network, 15 proteins, including Jun Proto-Oncogene (JUN), Decorin (DCN), Thrombospondin 1 (THBS1), Major Histocompatibility Complex, Class II, DR alpha (HLA-DRA), Endothelin 1 (EDN1), TIMP Metallopeptidase Inhibitor 3 (TIMP3), Transforming Growth Factor Beta 2 (TGFB2), 2′-5′-Oligoadenylate Synthetase 3 (OAS3), 2′-5′-Oligoadenylate Synthetase Like (OASL), 2′-5′-Oligoadenylate Synthetase 1 (OAS1), Platelet Derived Growth Factor Subunit B (PDGFB), Matrix Metallopeptidase 1 (MMP1), Early Growth Response 1 (EGR1), Syndecan 4 (SDC4) and Serpin Family E Member 1 (SERPINE1), were strongly connected to other proteins (degree centrality more than 8), indicating that they were hub genes (Fig. 6b). These hub genes might play crucial roles in olive oil-induced tumour growth. The hub genes and their corresponding degree are shown in Table 1. Moreover, the whole network was analysed utilizing the cytoscape plugin MCODE. The top 3 significant modules marked A, B and C, respectively, with a k-core more than 6, were selected as subnetworks, and 15 hub genes involved in the modules were marked with different colours (Fig. 7).

**Table 1:** Detail information of 15 hub genes between control diet and high olive diet selected by degree centrality

No.	Gene ID	Protein Names	Degree	Betweeness	Closeness
1	JUN	Transcription factor AP-1	17	12,790.9	0.0133
2	DCN	Decorin	13	5328.0	0.0132
3	THBS1	Thrombospondin-1	13	1587.0	0.0132
4	HLA-DRA	HLA class II histocompatibility antigen	13	4704.1	0.0131
5	TIMP3	Metalloproteinase inhibitor 3	11	1142.2	0.0132
6	TGFB2	Transforming growth factor beta-2	11	1907.1	0.0132
7	EDN1	Endothelin-1	11	4387.2	0.0132
8	PDGFB	Platelet-derived growth factor subunit B	10	1240.2	0.0132
9	OAS3	2′-5′-oligoadenylate synthase 3	10	1546.5	0.0131
10	OASL	2′-5′-oligoadenylate synthase-like protein	10	1546.5	0.0131
11	OAS1	2′-5′-oligoadenylate synthase 1	10	1546.5	0.0131
12	SDC4	Syndecan-4	9	2005.1	0.0132
13	SERPINE1	Endothelial plasminogen activator inhibitor	9	3693.0	0.0132
14	MMP1	Matrix metalloproteinase-1	9	2731.9	0.0132
15	EGR1	Early growth response protein 1	9	5957.4	0.0132

### Confirmation of DEGs by RT-qPCR

**Fig. 8:**
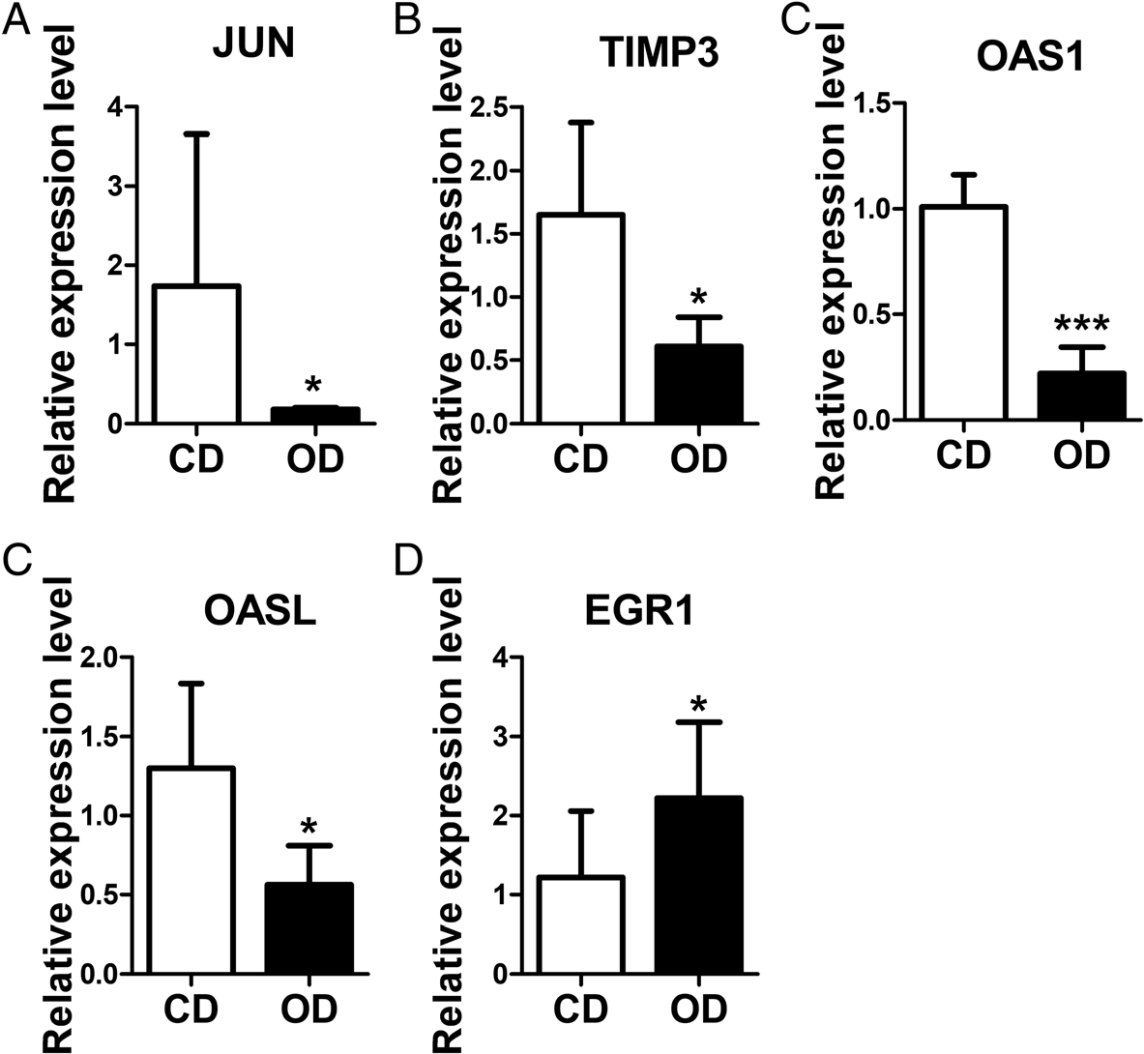
Real-time PCR analyses of DEGs. RNA sequencing shows the transcript levels of 5 genes. Relative levels of the transcript expression of EGR1 (up-regulated), JUN, TIMP3, OAS1 and OASL (down-regulated) in xenograft tumour tissues of the high olive oil group (*n* = 3) compared with those in the control diet group (*n* = 3). Comparisons among groups were determined using Student’s t-test. Significant differences were considered at *P < 0.05 and ***P < 0.001 versus control group. β-Actin was used as the reference gene

Five DEGs (JUN, TIMP3, OAS1, OASL, and EGR1) were arbitrarily selected to confirm their differential expression levels by RT-qPCR. As shown in Fig. 8, the qPCR results of the DEGs agreed with those of RNA-Seq analysis, suggesting the RNA-Seq data used in the present study were reliable and accurate. Both the OD group and CD group had three independent samples, and all the samples were processed in triplicate.

## Discussion

Olive oil, regarded as a key nutraceutical component of the traditional Mediterranean diet, exerts a beneficial impact mainly on the development and progression of cardiovascular risk events [32]. However, in the last two decades, there have been puzzling results regarding the possible role of dietary olive oil in cancer prevention and treatment. In the present study, we showed that feeding nude mice a high olive oil diet enhances xenograft tumour growth and progression. A bioinformatics analysis was performed to explore potential crucial DEGs associated with high olive oil diet. Then an advanced analysis was conducted to give insights into the biological functions and pathways involving DEGs. Finally, by building the PPI, we identified some hub genes and three subnetworks that might play decisive roles in the boosting effect of experimental CC.

As a result, 648 DEGs were screened out, including 155 up-regulated and 493 down-regulated DEGs. The up-regulated DEGs were mainly involved in functions related to positive regulation of transcription, such as EGR1 and Forkhead Box N2 (FOXN2). CC has been proven to be related to the disruption of transcription, including enhanced oncogene expression, loss of function of tumour suppressor genes and DNA repair genes [33]. EGR1, the only up-regulated hub gene in this study, activates expression of some cell cycle-related genes (e.g., cyclin D2 and Mitotic Arrest Deficient Protein 2) [34] and several growth factors (e.g., Platelet Derived Growth Factor Subunit A, Transforming Growth Factor Beta 1 and Insulin- Like Growth Factor 2) [35], suggesting its important role as an oncogene. EGR1 has been linked to some key cellular functions, such as proliferation [36] and migration [37]. Moreover, high levels of EGR1 were seen in CC tissues compared with that in normal tissues, suggesting its role in cervical oncogenesis [38]. The down-regulated DEGs were mainly related to the negative regulation of cell proliferation, such as Collagen Type IV Alpha 3 Chain and TGFB2. Furthermore, enriched KEGG pathways of up-regulated DEGs only included the Wnt signalling pathway. A disturbance in the Wnt signalling pathway has been highly noted in various cancers, including CC [39, 40]. Down-regulated DEGs were mostly associated with antigen processing and presentation. These altered genes and pathways may enhance our understanding of the mechanisms of dietary olive oil-induced tumour progression .

Through PPI network construction, a series of hub genes/proteins has been observed to form a local network, including JUN, DCN, THBS1, HLA-DRA, EDN1, TIMP3, TGFB2, OAS3, OASL, OAS1, PDGFB, MMP1, EGR1, SDC4 and SERPINE1, of which most DEGs have been reported to associate with cancer development. JUN encoding the c-Jun protein is among the hub genes that exhibit the highest degree of connectivity. JUN is widely regarded as an oncogene, which is the most extensively studied protein of the activator protein-1 (AP-1) complex and is involved in numerous cell activities, including proliferation, migration and tumour progression [41]. On the other hand, the c-Jun NH2-terminal kinase (JNK) /c-Jun pathway is an important pro-apoptotic signalling pathway in cancer cells. In our present study, JUN was significantly decreased in the xenografts of olive oil-fed mice. To address the possible role of JUN in CC, we analysed the cervical and endocervical cancers dataset of The Cancer Genome Atlas (TCGA) and found that the expression of JUN was also significantly down-regulated in CC tissues compared with that in normal tissues. The second hub gene, DCN, encoding Decorin, is markedly related to two main themes—maintenance of cellular structure and regulation of signal transduction pathways—culminating in anti-tumourigenic effects [42]. Studies have shown that deficiency of DCN is permissive for tumour development [43], whereas overexpression of DCN could block the cell cycle and decrease the invasive ability of cancer cells [42]. Although numerous applications of DCN as an anticancer therapeutic have been carried out, few studies have addressed the role of DCN in CC. THBS1, the third hub gene, is a 450-kDa homotrimeric matricellular glycoprotein with potent antiangiogenic effects by inhibiting the activity of Vascular Endothelial Growth Factor (VEGF) [44, 45]. Depend on its role to suppress angiogenesis, THBS1 was shown to retard tumour growth and is often found down-regulated in tumour samples [46]. In this study, we speculated that dietary high olive oil intake may contribute to tumourigenesis via JUN, DCN and THBS1-related signalling pathways, thereby maintaining proliferative capacity, anti-apoptosis, and promoting angiogenesis.

Subsequently, subnetwork analysis of the PPI network revealed that 3 clusters of genes representing a more topological central location were associated with CC proliferation and migration. Due to the inclusion of multiple OAS protein family members, the subnetwork that maintained the highest k-core value was associated with viral replication. The OAS proteins, consisting of OAS1/2/3 and OASL protein, were the first interferon-induced anti-viral proteins impeding the translation of viral nucleic acids [47]. The association between HPV and CC has been clearly established [48], and it is widely accepted that the major cause of CC is chronic infection with oncogenic HPV. However, the role of OAS family members during tumour development is not well documented.

Regarding the molecular mechanisms of olive oil and other dietary lipids on cancer, these aspects include: 1) influence on lipid peroxidation and the subsequent oxidative DNA damage, which can modulate inflammation and tumor development; 2) influence on transcription factor activity, gene expression, and signal transduction, which leads to changes in metabolism, cell proliferation, and angiogenesis; 3) alteration of the immune system, which leads to immune function abnormalities and inflammatory cytokine production imbalance [49–51].

Based on these clues, we try to find the potential links between dietary olive oil and the hub genes. Inflammation is a hallmark of cancer [52] and involved in all stages of the malignant process [53]. A high-fat diet can induce chronic inflammation, which in turn may aggravate oxidative stress and lipid peroxidation [54]. TIMP3, listed in the top centrality of down-regulated DEGs, has been shown to regulate TNF functions and modulate inflammatory responses [55]. In addition, the effects of dietary lipids and their metabolites on carcinogenesis may be directly mediated by binding to various nuclear receptors (PPAR, HNF4A) and activating their transcription factor action, or indirectly mediated as the result of changes in the abundance of regulatory transcription factors (SREBP, NFκB) [56]. Hub genes such as JUN and EGR1 are critical transcription factor, which can bind to NFκB and PPARγ respectively to regulate various gene expressions. Furthermore, immune system is critical for tumour control and immune destruction has been recognized as a new hallmark of tumorigenesis [57]. Dietary lipids, especially olive oil, have been shown to modulate the immune and inflammatory responses [58]. Recently, the OAS proteins are connected to innate immune-activated diseases and the activation of the OAS-RNase L axis has emerged as a feature of early immune transcriptome [59] [60]. However, there is no evidence that illuminate the relationship between olive oil and these hub genes. Our further work will conduct to establish the role of dietary olive oil in the control of these hub genes and their regulatory networks.

## Conclusions

In conclusion, our study suggests that high olive oil diet aggravates CC progression in vivo and in vitro. A comprehensive bioinformatics analysis showed distinct gene expression patterns between CD-fed mice and OD-fed mice and provides a set of useful hub genes for future investigation into molecular mechanisms and biomarkers.

## Additional files

Additional file 1: **Table S1.** Compositions of experimental diets (DOC 33 kb)

Additional file 2: **Table S2.** Primer sequences of the DEGs investigated in RT-qPCR analysis (DOC 32 kb)

## Abbreviations

AP-1: Activator protein-1
BP: Biological process
CC: Cellular component
CC: Cervical cancer
CD: Control diet
DAVID: Database for Annotation, Visualization and Integrated Discovery
DCN: Decorin
DMEM: Dulbecco’s modified Eagle’s medium
EDN1: Endothelin 1
EGR1: Early Growth Response 1
FBS: Foetal bovine serum
FC: Fold-change
FDR: False discovery rate
FOXN2: Forkhead Box N2
GO: Gene ontology
H&E: Haematoxylin and eosin
HLA-DRA: Major Histocompatibility Complex, Class II, DR Alpha
HPV: Human papillomavirus
JNK: c-Jun NH2-terminal kinase
JUN: Jun proto-oncogene
KEGG: Kyoto Encyclopedia of Genes and Genomes
MF: Molecular function
MMP1: Matrix Metallopeptidase 1
OA: Oleic acid
OAS1: 2′-5′-Oligoadenylate Synthetase 1
OAS3: 2′-5′-Oligoadenylate Synthetase 3
OASL: 2′-5′-Oligoadenylate Synthetase Like
OD: Olive oil diet
PCNA: Proliferating cell nuclear antigen
PDGFB: Platelet Derived Growth Factor Subunit B
PPI: Protein-Protein Interaction
RPKM: Reads per kilobase per million
RT-qPCR: Real-time quantitative PCR
SDC4: Syndecan 4
SERPINE1: Serpin Family E Member 1
STRING: Search Tool for the Retrieval of Interacting Genes
TCGA: The Cancer Genome Atlas
TGFB2: Transforming Growth Factor Beta 2
THBS1: Thrombospondin 1
TIMP3: TIMP Metallopeptidase Inhibitor 3
VEGF: Vascular Endothelial Growth Factor
