# Supplementary material for: High olive oil diets enhance cervical tumour growth in mice: transcriptome analysis for potential candidate genes and pathways
Source: Lipids Health Dis. 2019 Mar 28;18:76. doi: 10.1186/s12944-019-1023-6 (PMC6440132; doi:10.1186/s12944-019-1023-6)
Supplement: Supplementary file 1 — Table S1. Compositions of experimental diets (DOC 33 kb) [file 12944_2019_1023_MOESM1_ESM.doc]

**Additional file 1**

**Table S1. Compositions of experimental diets.**

|  | **Control Diet**  **(10 kcal % Fat)** | | **High-Olive Oil Diet**  **(45 kcal % Fat)** | |
| --- | --- | --- | --- | --- |
| **Producte#** | **D12450B** | | **OD17010901** | |
| Ingredient  Casein, 80 Mesh  L-Cystine  Corn Starch  Maltodextrin 10  Sucrose  Cellulose, BW200  Soybean Oil  Lard*  Olive Oil Mineral Mix S10026  DiCalciumPhosphate  Calcium Carbonate  Potassium Citrate,1H2O  Vitamin Mix V10001  Choline Bitartrate  FD&C Yellow Dye #5  Total | g  200  3  315  35  350  50  25  20  0  10  13  5.5  16.5  10  2  0.05  1055.05 | Kcal  800  12  1260  140  1400  0  225  180  0  0  0  0  0  40  0  0  4057 | g  200  3  72.8  100  172.8  50  25  0  177.5  10  13  5.5  16.5  10  2  0  858.1 | Kcal  800  12  291  400  691  0  225  0  1598  0  0  0  0  40  0  0  4057 |
