# Supplementary material for: High olive oil diets enhance cervical tumour growth in mice: transcriptome analysis for potential candidate genes and pathways
Source: Lipids Health Dis. 2019 Mar 28;18:76. doi: 10.1186/s12944-019-1023-6 (PMC6440132; doi:10.1186/s12944-019-1023-6)
Supplement: Supplementary file 2 — Table S2. Primer sequences of the DEGs investigated in RT-qPCR analysis (DOC 32 kb) [file 12944_2019_1023_MOESM2_ESM.doc]

**Additional file 2**

**Table S2. Primer sequences of the DEGs investigated in RT-PCR analysis.**

| **Gene** | **Primer sequences** |
| --- | --- |
| β-Actin | F 5′-AACGGCTCCGGCATGTGCAA-3′ R 5′-CTTCTGACCCATGCCCACCA-3′ |
| JUN | F 5′-TCCAAGTGCCGAAAAAGGAAG-3′  R 5′-CGAGTTCTGAGCTTTCAAGGT-3′ |
| TIMP3 | F 5′-CATGTGCAGTACATCCATACGG-3′  R 5′-CATCATAGACGCGACCTGTCA-3′ |
| OAS1 | F 5′-TGTCCAAGGTGGTAAAGGGTG-3′  R 5′-CCGGCGATTTAACTGATCCTG-3′ |
| OASL | F 5′-CCATTGTGCCTGCCTACAGAG-3′  R 5′-CCATTGTGCCTGCCTACAGAG-3′ |
| EGR1 | F 5′-GGTCAGTGGCCTAGTGAGC-3′  R 5′-GTGCCGCTGAGTAAATGGGA-3′ |
